# Implementing modern teaching approaches for evidence-based practice in German midwifery university education: a qualitative study of educator experiences, facilitators, and barriers

**DOI:** 10.1186/s12909-026-10807-5

**Published:** 2026-11-06

**Authors:** Grace Komuhangi, Florian Neuhann, Jürgen Wacker, Brian Mikka, Charlotte Fery, Valerie R. Louis

**Affiliations:** 1 Faculty of Medicine and University Hospital, and Medical Faculty, Heidelberg Institute of Global Health (HIGH), Heidelberg University, Im Neuenheimer Feld 130.3, Heidelberg, Baden Wüttemberg 69120 Germany; 2 Department of Obstetrics and Gynecology, Faculty of Medicine, Heidelberg University, Im Neuenheimer Feld 130.3, Heidelberg, 69120 Germany; 3 Department of Clinical Epidemiology, College of Health Sciences, School of Medicine, Makerere University, P.O. Box 7072, Kampala, Uganda; 4 Midwifery Research and Education Unit, Department of Obstetrics, Gynecology, and Reproductive Medicine, Hannover Medical School, OE 6410, Carl-Neuberg-Str. 1, Hannover, 30625 Germany

**Keywords:** Evidence-based practice, Midwifery education, Qualitative research, Competency-based education, Germany, Academisation, Hebammenreformgesetz

## Abstract

**Background:**

Germany now mandates the transition of midwifery education from vocational training to university-based bachelor’s programmes, as required by the Hebammenreformgesetz (2020). This reform necessitates teaching approaches that emphasise competency in Evidence-Based Practice (EBP). However, limited information exists regarding midwifery educators’ experiences, the barriers encountered, and the facilitators supporting this transition. This study examined midwifery educators’ experiences implementing contemporary EBP teaching strategies in German universities, as well as the facilitators and barriers they faced.

**Methods:**

We used an exploratory descriptive qualitative design. Eleven midwifery educators from five universities and universities of applied sciences across three German states participated in interviews. Data were analysed using inductive, team-based thematic analysis in accordance with Braun and Clarke’s six-phase framework. After the inductive analysis, Competency-Based Education (CBE) theory and Social Cognitive Learning Theory (SCLT) were applied as interpretive frameworks.

**Results:**

Three primary themes were identified. First, educators reported experiences with modern teaching, encompassing new curriculum design, active-learning strategies, and increased academic freedom. Second, barriers included institutional constraints, insufficient educator skills, a disconnect between theory and practice, and student resistance. Third, facilitators included strong infrastructure, robust professional networks, and perceived improvements in student learning.

**Conclusions:**

Midwifery educators in Germany are implementing modern EBP teaching methods despite significant challenges. Participants indicated that structured faculty development, formalised partnerships between universities and clinical sites, and a standardised national description of EBP competencies may support the sustainability of these changes. These recommendations require evaluation in larger and more representative samples. Future research should incorporate perspectives from students and clinical supervisors and employ validated instruments to assess the relationship between education and practice outcomes.

**Supplementary Information:**

The online version contains supplementary material available at https://doi.org/10.1186/s12909-026-10807-5.

## Background

### Evidence-based practice and EBP competencies

Evidence-Based Practice (EBP) integrates the best available research evidence with clinical expertise and individual patient values [1]. In midwifery, skilled birth attendants use EBP to improve maternal and neonatal outcomes [2]. Evidence-based maternity care is an important component of improving maternal and perinatal outcomes [3]. The International Confederation of Midwives (ICM) 2024 Essential Competencies designate EBP competencies, including evidence retrieval, critical appraisal, and evidence application, as fundamental to safe and effective midwifery practice [4].

Competency-Based Education (CBE) theory provides the pedagogical foundation for EBP instruction, building on the central importance of EBP. CBE organises learning around clearly defined and measurable competency outcomes, rather than focusing exclusively on knowledge transmission [5, 6]. In healthcare education, curricula are designed to progressively develop students’ abilities to identify clinical questions, retrieve and appraise evidence, and apply findings in practice [7].

### Modern teaching approaches in midwifery education

Educators foster EBP competencies through modern teaching approaches, including Problem-Based Learning (PBL), simulation-based learning, case-based learning, blended learning, and experiential learning [8–10]. These methods share a constructivist orientation, positioning students as active participants in knowledge construction rather than passive recipients [11]. Additionally, SCLT emphasises observational learning, self-efficacy, and reciprocal determinism, providing a complementary framework for understanding how educators model EBP behaviours and how students develop confidence in EBP application [12, 13].

Most evidence regarding these teaching approaches originates from nursing, medicine, and allied health education, where studies have primarily evaluated their impact on students’ EBP knowledge, attitudes, and skills [8–10]. However, understanding remains limited about how educators implement these methods in midwifery degree programmes, including their working conditions, available support, and encountered obstacles [10, 14]. Furthermore, current research does not sufficiently address the specific challenges educators face when implementing modern EBP teaching approaches during Germany’s ongoing transitional period, nor does it examine the facilitators and barriers institutions encounter when embedding EBP competency-oriented curricula in this distinct context.

### German midwifery education context

In this context, the Hebammenreformgesetz (HebRefG) of 2019 (effective 2020) mandates the academisation of midwifery education in Germany, representing a fundamental paradigm shift [15]. This legislation transitions midwifery education from hospital-based vocational training to university-level bachelor’s degree programmes, typically organised as dual-programme models that integrate theoretical and practical training [16, 17]. Vocational training could still commence until the end of 2022 and must be completed by the end of 2027. Thereafter, all newly qualifying midwives in Germany will be required to hold a bachelor’s degree, necessitating a transformation in how educators conceptualise and deliver EBP instruction [18].

The reform implements Directive 2013/55/EU, which amended Directive 2005/36/EC concerning the recognition of professional qualifications [19]. The Midwifery Act and the Study and Examination Regulations for Midwives (HebStPrV) require graduates to practise based on current scientific knowledge [15, 20]. Consequently, EBP is not an optional component of academic midwifery education but a defining objective, consistent with the ICM Essential Competencies [4]. The transition to university-based education is expected to support EBP learning in three primary ways: students are instructed by educators with research training; they systematically learn research methods and critical appraisal within a curriculum designed to build EBP competencies progressively; and they study in an environment where research is both produced and utilised [21, 22]. Research-inspired teaching, in which students participate in research processes, exemplifies this connection [23]. Several approaches examined in this study, such as problem-based learning and spiral curricula that progress from research question to appraisal and application, are grounded in the same principle.

This significant legislative transition presents educators with substantial challenges. Many educators completed their professional training under the vocational model and must now teach within an academic framework while developing EBP pedagogical competencies that may not have been part of their original preparation [24, 25]. At the same time, the reform introduces new opportunities, as the university context provides access to academic infrastructure, research communities, and curricular autonomy previously unavailable in the vocational model [21]. The realisation of anticipated benefits for EBP learning depends largely on how programmes and educators navigate this transition. Insufficient support during this process may result in graduates entering practice without the EBP competencies intended by the reform, potentially affecting the quality of maternity care [3].

### Theoretical framework

CBE and SCLT were applied as post hoc interpretive frameworks following the inductive analysis of the findings. CBE theory [5, 6] provides a structure for understanding how educators design curricula aligned with EBP competency outcomes. SCLT [12] elucidates the social and observational processes in pedagogical learning, including the development of new teaching behaviours through peer observation, vicarious reinforcement, and self-efficacy. These frameworks serve to interpret and contextualise the findings within the broader literature, rather than to guide the initial analysis.

### Study aim and research questions

In light of the policy context and the identified evidence gap, this study examines the experiences, facilitators, and barriers associated with the use of modern teaching approaches for EBP competency development. The focus is on undergraduate midwifery educators at German universities. The research questions are as follows:

1. What are the experiences of midwifery educators with modern teaching approaches for EBP in German universities?
2. What are the facilitators that support the implementation of modern teaching approaches for EBP in midwifery education?
3. What are the barriers that hinder the implementation of modern teaching approaches for EBP in midwifery education?

## Methods

### Study design

An exploratory descriptive qualitative design was employed. This approach aligns with the aim of empirically grounding educators’ experiences without imposing strong a priori interpretive structures [26]. This design was selected instead of phenomenological, grounded theory, or ethnographic methods, which emphasise lived-experience meanings (phenomenology), theory generation (grounded theory), or cultural immersion (ethnography) [27]. In contrast, the research questions in this study sought descriptive accounts of implementation experiences, as well as the identification of facilitators and barriers.

### Setting and participants

Purposive maximum-variation sampling was used to recruit midwifery educators from German universities and universities of applied sciences (Fachhochschulen). These institutions had established undergraduate midwifery programmes following the HebRefG reform. Maximum-variation sampling was selected to capture diverse perspectives across institution types and programme maturity [28]. The inclusion criteria were: (1) employment in a teaching role in an accredited undergraduate midwifery programme in Germany; (2) responsibility for teaching at least one module with explicit EBP content; and (3) programme establishment between 2019 and 2022. Exclusion criteria included educators whose programmes had not yet admitted their first student cohort or who could not participate in a German- or English-language interview. The principal investigator recruited participants through professional networks within the German midwifery academic community, including the German Midwifery Academic Society (Deutsche Gesellschaft für Hebammenwissenschaft, DGHWi). Potential participants were invited by email and provided with a study information sheet prior to consenting. The numbers invited, responded, and did not respond are not reported; therefore, a recruitment rate cannot be calculated.

Five institutions from three federal states (Bavaria, Rhineland-Palatinate, and Lower Saxony) were represented (Table 1). A total of 11 educators participated: 5 from Bavaria, 2 from Rhineland-Palatinate, and 4 from Lower Saxony, with no more than 3 from any single institution. Participants held a range of roles, including research assistant, professor, and programme director (Table 2). The sample size was not predetermined. Recruitment concluded after 11 interviews because we could not recruit additional participants. This stopping decision was pragmatic rather than based on a prospectively documented saturation threshold. During coding and theme refinement, the analysis compared accounts across the full dataset and reviewed candidate themes against both supporting and divergent extracts. We assessed sample adequacy by information power rather than a claim of saturation. The adequacy of the sample was further appraised against the five dimensions of information power proposed by Malterud et al. [29]: the study aim was narrow, focusing on EBP teaching within a single professional group in one country; sample specificity was high, as all participants taught EBP content on accredited undergraduate midwifery programmes; the interview guide was grounded in the literature on EBP education and CBE; dialogue quality was strong, with interviews of 45 to 90 min conducted in German by an interviewer who shared the participants’ professional background; and the analysis compared accounts across participants rather than examining single cases in depth. Collectively, these features support the adequacy of a sample of 11 for a descriptive study.

**Table 1:** Participating institutions (5 institutions, 11 participants). Institution names are anonymised

Institution (anonymised)	Federal State	Institution Type	Programme Established	Participants (*N*)
University A	Bavaria	University of Applied Sciences (public)	2019	2
University B	Bavaria	University (public)	2019	3
University C	Rhineland-Palatinate	University of Applied Sciences (private)	2021	2
University D	Lower Saxony	University of Applied Sciences (public)	2021	2
University E	Lower Saxony	University (public)	2020	2
Total				11

### Data collection

We collected data through semi-structured, in-depth individual interviews (IDIs), conducted face-to-face at each participant’s institution between January and June 2025. The principal investigator (PI) developed the interview guide following a review of literature on EBP education, competency-based education, and qualitative health research. The supervisory team reviewed and approved the guide prior to its use. The guide addressed four themes: (1) experiences with EBP teaching approaches; (2) perceived facilitators of implementation; (3) perceived barriers to implementation; and (4) recommendations for strengthening EBP competency development. Example prompt questions included: ‘Can you describe how you currently teach EBP in your module?‘; ‘What supports you in implementing active learning approaches?‘; ‘What obstacles have you encountered in embedding EBP into your teaching?‘; and ‘How do you assess students’ EBP competencies?’ We used flexible probing to encourage elaboration and to explore emerging issues.

We conducted interviews in German and audio-recorded them with participants’ consent. Each interview lasted between 45 and 90 min. After each session, the PI recorded brief field notes capturing contextual observations and initial analytic impressions. The interview guide was not formally piloted; however, we refined it after the first two interviews based on the PI’s reflections regarding question clarity and participant responses.

### Transcription and translation

The PI transcribed all audio recordings verbatim in German to preserve linguistic nuance and idiomatic expression. We reviewed transcripts for accuracy against the audio recordings. The PI, a bilingual German-English speaker, translated selected transcript segments into English for reporting, and a second bilingual research team member cross-checked these translations. To minimise meaning loss, the translation team discussed and documented disputed translations, and transcript review and thematic analysis were conducted in German. We finalised English translations of representative quotations only after theme development was complete, ensuring analytical decisions were grounded in the source-language data.

### Data analysis

We analysed the data using inductive, team-based thematic analysis, following the six phases described by Braun and Clarke [30]. We do not describe our approach as reflexive thematic analysis, as our procedures included independent coding and agreement checks, which Braun and Clarke distinguish from the reflexive approach [31]. Our epistemological position was pragmatic: we treated participants’ accounts as meaningful reports of their teaching experience, while recognising that these accounts were shaped by the interview context and by our own interpretation. The process included: (1) familiarisation with the data through repeated reading of transcripts and field notes; (2) generation of initial codes applied inductively to all transcripts; (3) searching for themes by collating codes into candidate themes; (4) reviewing and refining themes against the coded extracts and the full dataset; (5) defining and naming themes; and (6) producing the final report. We conducted the analysis using OpenCode version 4.03.

The PI and a second researcher independently coded the first four transcripts. Percentage agreement in applying the initial codes to these transcripts exceeded 85% [32]. This check addressed only consistent code application; it identified unclear code definitions and prompted discussion, rather than measuring theme validity. We resolved discrepancies through discussion and consensus, which led to refined code definitions. When disagreements persisted, the team consulted a third member. The PI then coded the remaining transcripts using the refined codebook. Ongoing peer debriefing discussions with the supervisory team reviewed emerging interpretations and prevented premature closure. We maintained reflexive memos throughout. The PI applied CBE and SCLT as post hoc interpretive lenses in the Discussion, after inductive theme development was complete. These conceptual models were not used as a priori coding categories.

We developed themes from the coded data through an iterative process. The PI compared codes across transcripts and grouped related codes into candidate themes. Each candidate theme was reviewed against its supporting extracts, extracts that did not fit or suggested alternative interpretations, and the complete transcripts to ensure it represented the data as a whole. We discussed candidate themes and their boundaries in peer debriefing meetings with the supervisory team before defining and naming them, resulting in the thematic structure shown in Table 3. We recorded analytic decisions in the audit trail.

### Methodological rigour and trustworthiness

Several strategies were employed to ensure the trustworthiness of the findings, specifically credibility and dependability [33]. Credibility, defined as confidence in the findings, was strengthened through prolonged engagement with the data, peer debriefing, and member checking, in which participants verified the researchers’ interpretations. During member checking, participants reviewed summary interpretations of their interviews and either confirmed or corrected the researchers’ interpretations. The maintenance of an audit trail, consisting of detailed records of research steps, further contributed to credibility. Transferability, or the potential for findings to be applicable in other contexts, was supported by providing detailed descriptions of the study context, setting, and participants. Dependability, referring to the stability of findings over time, was enhanced by the audit trail documenting analytic decisions and methodological changes. Confirmability, the extent to which findings are shaped by participants rather than researcher bias, was promoted through reflexive journaling and a documented coding consensus process.

### Reflexivity

The PI is a qualified midwife with more than 20 years of international experience in midwifery practice and education, and currently holds a teaching position in German higher education. This professional background facilitated rapport with participants and provided deep situational understanding. However, it also increased the risk of over-identification with participants’ perspectives and confirming anticipated findings. These risks were addressed through reflexive journaling during data collection and analysis. Peer debriefing with supervisors from diverse disciplinary and experiential backgrounds further mitigated these concerns. During coding meetings, the PI and research team explicitly reflected on how the PI’s assumptions might influence interpretive decisions. The PI acknowledged the prior professional relationship between the PI and some participants as a potential source of social desirability bias. Participants were informed that candid, including critical, responses were both welcome and essential to the study.

### Ethics

#### Ethical considerations

Ethical approval was obtained from the Ethics Committee of the Medical Faculty, Heidelberg University (Reference S-709/2024, December 23, 2024). All participants provided written informed consent after being fully informed about the study. The research complied with the Declaration of Helsinki and Article 13 of the General Data Protection Regulation (GDPR). We transcribed audio recordings verbatim, pseudonymised them by replacing identifying details with codes, and permanently deleted them after verifying the transcription.

#### Data Availability

The datasets generated and analysed in this study are not publicly available due to confidentiality protections required by the study’s ethical approval. Anonymised transcripts and the codebook may be made available to qualified researchers upon reasonable request to the corresponding author, subject to applicable data protection regulations.

## Results

### Participant characteristics

Eleven midwifery educators from five institutions in three federal states participated in the study. Table 2 presents the participant characteristics. The sample exhibited demographic and professional diversity, with ages ranging from 40 to 55 years (median 48) and teaching experience from 1 to 19 years (median 5). Most participants held a master’s degree (*n* = 9), while two held doctorates. Reported roles included professor, lecturer, scientific employee, research assistant, practice coordinator, and programme director. All participants were actively engaged in evidence-based practice (EBP) teaching at the time of the interview. Systematic data on prior pedagogical and formal EBP training were not collected; however, many participants reported entering academic teaching without formal pedagogical preparation (ST 2.2). Descriptive statistics were used to characterise the sample. Inferential statistical analyses were not performed.

**Table 2:** Participant characteristics (*N* = 11)

Variable	Categories	Frequency (*N* = 11)
Age (years)	Median (Range)	48 (40–55)
Highest qualification	Doctorate	2
	Master’s degree	9
Current role	Professor	2
	Lecturer	3
	Scientific employee	2
	Research assistant	2
	Practice coordinator	1
	Programme director	1
Teaching experience (years)	Median (Range)	5 (1–19)

Data are presented as median (range) for continuous variables and as frequency (n) for categorical variables. No data were missing for the characteristics presented in Table 2

Analysis of the data identified three main themes and eleven sub-themes (Table 3). Each theme is introduced with its central finding and supported by representative quotations. The phrase ‘at all 5 institutions’ indicates that at least one educator from each institution reported the observed pattern. Application of the CBE and SCLT frameworks is addressed in the Discussion.

**Table 3:** Summary of themes, sub-themes, and representative codes

Theme	Sub-theme	Representative Codes
Theme 1 Experiences with Modern Teaching Approaches	ST 1.1: Revolutionary shift from traditional training	Self-directed pedagogical learning; transition from rote-learning culture; reorientation of professional identity; challenge to internalised vocational norms
	ST 1.2: Innovative curriculum design	Spiral curriculum (progressive competency revisiting); collaborative module design; deliberate EBP sequencing across semesters; theory–skills–practice alignment
	ST 1.3: Creative and diverse teaching approaches	Problem-based learning (PBL); case-based learning; simulation; flipped classroom; gamification (escape rooms, Lego Serious Play); virtual reality integration
	ST 1.4: Academic freedom and pedagogical autonomy	Constitutional Lehrfreiheit; individual methodological autonomy; experimental pedagogy; personal teaching philosophy guiding method selection
Theme 2 Implementation Barriers	ST 2.1: Systemic institutional barriers	Time pressure; compressed study schedules; financial constraints; bureaucratic procurement; staff shortages; inadequate infrastructure
	ST 2.2: Educator competency gaps	Abrupt clinician-to-academic transition; absence of formal pedagogical training; reliance on textbooks; self-taught teaching skills; limited assessment design experience
	ST 2.3: Theory–practice divide	Contradiction between university EBP standards and clinical norms; student confusion; clinical mentor resistance; parallel vocational–academic workforce; institutional bridging attempts
	ST 2.4: Student resistance and learning challenges	Academic writing overload; resistance to group work; preference for frontal instruction; passive learning expectations from prior vocational socialisation
Theme 3 Implementation Facilitators	ST 3.1: Robust resource infrastructure	Library and database access; e-journal licensing; skills laboratories; simulation centres; VR and ultrasound technology; institutional funding for innovation
	ST 3.2: Strong collaborative networks	Interdisciplinary co-teaching; national midwifery academic society networks; peer knowledge-sharing; academic–clinical partnership building
	ST 3.3: Student learning transformation	Observable critical thinking development; evidence-seeking in clinical placement; students questioning non-EBP practice; growing EBP professional identity

*EBP* evidence-based practice, *PBL* problem-based learning, *IDI* in-depth interview. Representative codes are illustrative examples from the coding set, not an exhaustive list

### Theme 1: Experiences with modern teaching approaches

#### ST 1.1: Revolutionary shift from traditional training

Educators described a fundamental personal and professional transformation as they shifted into university-based EBP teaching. They reported a strong need for self-directed pedagogical learning. Educators taught themselves new methods they had not previously learned.

Several educators described dismantling deeply internalised vocational teaching norms:

*“I had to completely unlearn how I was taught. In the hospital*,* we followed protocol. Now I have to help students question the protocol*,* and I had to learn to question it myself first. (P03)”*.

Educators from the programmes established in 2019 said they had more time to build teaching confidence, while those in newer programmes reported greater uncertainty. Still, regardless of how long programmes had been established, everyone described a shift in identity from clinician to academic teacher.

#### ST 1.2: Innovative curriculum design

Across all five institutions, educators developed spiral curricula that repeatedly addressed EBP competencies, each time with greater complexity as semesters progressed. Notably, this consistency emerged even though no national curriculum specified such an approach:

*“We mapped EBP across all eight semesters. We began with a research question*,* then moved to literature searching*,* followed by critical appraisal*,* synthesis*,* and finally application in practice. As a result*,* each semester builds on the one before. (P07) “*.

Educators also described deliberately sequencing theory, skills, laboratory practice, and clinical placement EBP activities to reduce the gap between academic learning and clinical application. Collaborative module design across teaching teams was reported as a facilitator of this curriculum coherence.

#### ST 1.3: Creative and diverse teaching approaches

Educators reported using a range of active and experiential learning methods to develop EBP competencies. PBL was the most frequently reported method and was used at all 5 institutions. Case-based learning, simulation and the flipped classroom followed, in that order of frequency. Several educators also described gamification strategies, such as escape rooms and Lego Serious Play (Table 3).

Two institutions used virtual reality for clinical-scenario simulation, mainly for obstetric emergency training with embedded EBP decision-making. Educators initially observed student reluctance. As students adapted, their engagement increased (see ST 2.4).

#### ST 1.4: Academic freedom and pedagogical autonomy

Educators identified the constitutional principle of Lehrfreiheit (academic freedom of teaching) as both enabling and, at times, challenging. On the enabling side, freedom of teaching permitted educators to select, adapt, and combine teaching approaches based on their own professional judgement, without requiring institutional approval for each methodological decision:

*“I have complete freedom in how I teach. If I want to try a new method*,* I try it. There is no one to say no. This is very different from the hospital*,* where everything has to go through governance. (P09)”*.

However, several educators noted that this autonomy, in the absence of shared pedagogical standards or professional development support, could result in inconsistent EBP teaching quality across modules and institutions.

### Theme 2: Implementation barriers

#### ST 2.1: Systemic institutional barriers

The most consistently reported barriers were systemic and structural. Time pressure was mentioned by all 11 participants as a significant constraint: compressed study schedules, large module loads, and administrative burdens left little time for pedagogical development, preparation of innovative teaching materials, or reflection on practice.

*“The semester is 16 weeks. I have to cover the full midwifery research methods curriculum in that time*,* prepare all the materials*,* supervise practical sessions*,* mark assessments*,* attend meetings. There is no time to innovate. You do what you can manage. (P02)”*.

Financial constraints and bureaucratic procurement processes were reported at four of the five institutions as limiting access to simulation technology, software licences, and digital learning resources. Staff shortages, particularly at newer programmes with smaller faculties, meant that individual educators carried disproportionately large teaching loads.

#### ST 2.2: Educator competency gaps

A recurring finding was that many educators felt unprepared for university-level EBP instruction, particularly in teaching research methodology, designing academic assessments, and utilising digital teaching technologies. They often reported an abrupt shift from clinical midwifery practice to academic teaching, typically without formal pedagogical training.

*“No one taught me how to teach. I studied midwifery*,* not pedagogy. So*,* everything*,* how to facilitate a seminar*,* how to construct an assessment*,* how to deliver feedback*,* I had to learn myself: from books*,* colleagues*,* and through trial and error. (P11)”*.

Reliance on textbooks as primary teaching resources, limited experience with formative assessment design, and self-taught digital teaching skills were reported at all institutions. Educators with doctoral qualifications (*n* = 2) reported somewhat greater confidence in research methods teaching, though they also described gaps in practice-based EBP pedagogy.

#### ST 2.3: Theory–practice divide

A persistent finding across all institutions was tension between the EBP standards promoted in the university curriculum and the evidence-based practice norms (or their absence) encountered by students during clinical placements. Students regularly reported to educators that their clinical mentors dismissed academic EBP skills as impractical or irrelevant to clinical reality.

*“Students come back from placement and say: ‘My mentor told me we do not need evidence*,* we do it this way because it has always worked.’ It is very hard to maintain student motivation for EBP when the clinical world contradicts everything we teach. (P06)”*.

Educators at three institutions described attempting to address this divide through bridging initiatives, including inviting clinical supervisors to participate in EBP seminars and developing joint university–practice EBP competency frameworks. These efforts were reported as valuable but insufficiently resourced and not systematically supported at the institutional level.

#### ST 2.4: Student resistance and learning challenges

Educators at all institutions reported encountering initial student resistance to active and self-directed learning methods, attributing this primarily to prior socialisation into passive learning during vocational training. Students were described as frequently requesting frontal lecturing, expressing discomfort with group work, and struggling with academic writing demands.

*“In the first semester*,* they always ask: ‘Why can’t you just tell us the answer? Why do we have to search for it ourselves?’ They are used to being told what to do. This is a culture change*,* not just a teaching method change. (P04)”*.

Educators reported that this resistance typically diminished over the course of the programme as students gained confidence in self-directed EBP processes, though the pace of this shift varied considerably across individual students.

### Theme 3: Implementation facilitators

Alongside these barriers, educators identified conditions that helped them sustain EBP teaching. Several facilitators were the counterpart of barriers reported in Theme 2: resources and dedicated funding for innovation, for example, were available at some institutions and lacking at others.

#### ST 3.1: Robust resource infrastructure

Educators at well-resourced institutions identified access to comprehensive library and database infrastructure, including institutional e-journal subscriptions, academic database licences (e.g., CINAHL, Cochrane Library, PubMed), and dedicated skills laboratories, as important facilitators of EBP teaching.

*“We have a skills laboratory with high-fidelity simulation equipment*,* VR stations*,* and a library that is fully integrated into the building. Students can move from simulation to library research in the same session. This makes embodied EBP learning possible. (P08)”*.

Institutional funding specifically designated for teaching innovation was reported at two institutions and described as enabling educators to pilot and develop novel EBP learning activities. Lack of such dedicated funding was correspondingly identified as a barrier at the remaining three institutions.

#### ST 3.2: Strong collaborative networks

Collaborative relationships within institutions and across the emerging national German midwifery academic community were identified as key facilitators. Within institutions, interdisciplinary co-teaching, especially with colleagues from nursing, health sciences, and research methods, enriched EBP teaching and shared the preparatory workload.

*“I co-teach the evidence-based practice module with a nursing academic who has a PhD in health research. She brings the methodological expertise; I bring the midwifery context. Together we can offer students something neither of us could alone. (P01)”*.

At the national level, educators described the German midwifery academic society networks as providing peer support, opportunities for sharing teaching materials and assessment designs, and a forum for collective advocacy on curriculum standards. Several educators credited these networks with preventing professional isolation during the challenging early years of programme development.

#### ST 3.3: Student learning transformation

Despite the barriers described above, educators across all five institutions reported observing meaningful transformations in students’ EBP engagement over the course of their programmes. Observable indicators included critical questioning of clinical practices during placements, independent evidence-seeking to address clinical questions, and articulation of an emerging evidence-based professional identity.

*“By the third year*,* I see completely different students. They question things. They go to the evidence when they do not know something. They come back from placement and say*,* ‘I challenged my mentor and looked it up*,* and I was right.’ That is the whole point. (P10)”*.

Educators described this transformation as the most rewarding aspect of their work and as providing motivational resilience in the face of institutional and systemic barriers. Student transformation was also seen as a prospective mechanism for long-term cultural change in clinical settings, as newly qualifying midwives carry EBP values into the workforce.

## Discussion

### Summary of key findings

This qualitative study examined the implementation of contemporary EBP teaching approaches by midwifery educators following Germany’s academic reform of midwifery education. Three inductively derived themes emerged. First, educators reported significant pedagogical transformation and innovation. Second, systemic institutional constraints, competency gaps, and the persistent theory–practice divide were identified as barriers. Third, infrastructure, collaborative networks, and educators’ perceptions of student transformation were described as facilitators. These findings indicate that German midwifery educators exhibit substantial pedagogical creativity in implementing EBP teaching within a structurally complex environment. Nevertheless, ongoing barriers may impede the sustained integration of EBP competencies into the reformed curriculum.

### Interpretive discussion

When viewed through the lens of CBE theory, the independent yet convergent adoption of spiral curriculum structures across all five participating institutions is particularly noteworthy. This development has occurred in the absence of a national prescriptive framework. It is consistent with the CBE principle that EBP competency development requires iterative and scaffolded engagement rather than isolated instruction [5, 6]. Educators appear to have adopted this structural approach through professional reasoning and peer exchange, rather than through top-down mandates. While the data do not establish causality, one possible explanation is that CBE-aligned thinking is disseminating through informal networks within the emerging German midwifery academic community, even without formal CBE training. Although participants reported this convergence, the mechanisms underlying it were not directly examined. Additionally, the sample may overrepresent educators already engaged in innovation, potentially reflecting early adopters rather than the broader educator population. This interpretation remains tentative due to the small, purposive sample and should not be generalised beyond the participating institutions.

From the perspective of SCLT [12], educators reported significant pedagogical self-transformation. They engaged in self-directed learning of new teaching methods, observed colleagues, and gradually developed academic self-efficacy in teaching. These developments may reflect key SCLT mechanisms, such as vicarious reinforcement and self-regulated learning. The collaborative networks identified as facilitators (ST 3.2) may serve as communities of practice consistent with SCLT, offering opportunities for vicarious learning and social reinforcement of innovative pedagogical behaviours.

The ongoing theory-practice divide (ST 2.3) aligns with international evidence regarding barriers to implementing EBP in nursing and health professions education. Clinician scepticism, institutional culture [34], and the lack of practice-level EBP mentoring are consistently identified as significant obstacles [35–38]. A recent mixed methods systematic review of 59 studies in midwifery and nursing education similarly highlighted the importance of frameworks that bridge theory and practice, as well as collaborative partnerships, in connecting teaching, research, and EBP [22]. Participants’ accounts suggest that this divide may be sustained by differences between university EBP teaching and established clinical practices within Germany’s dual-programme model. Although educators described efforts to bridge this gap, they also reported limited institutional support for these initiatives.

The wider pattern of facilitators and barriers is also consistent with international evidence. Our systematic review of problem based learning for EBP teaching found that faculty preparation, adequate time, technological resources and organisational support favoured implementation, while limited infrastructure and faculty workload were common barriers [10]. The present study adds that in Germany these conditions are shaped by a legislative transition that is still under way.

SCLT also helps explain why the theory practice divide was so persistent in participants’ accounts. The theory holds that learners acquire behaviours largely by observing models whose actions are rewarded [12]. Participants described students who learned to appraise evidence at university and then observed clinical mentors who dismissed EBP as irrelevant to clinical reality (ST 2.3). In these settings, the most visible and credible models demonstrate the opposite of what is taught. Our findings extend this perspective by suggesting that educators’ own self efficacy shapes what students can observe: educators who described gaps in their research methods and pedagogical preparation (ST 2.2) were, in effect, modelling EBP teaching while still learning it themselves, whereas those with doctoral qualifications reported greater confidence. One interpretive possibility, which the data suggest but cannot demonstrate, is that the facilitator of student learning transformation (ST 3.3) reflects the SCLT concept of outcome expectations: educators’ reports of students questioning practice and seeking evidence appeared to reinforce their commitment to these approaches in the face of barriers [13]. These are educators’ perceptions of student learning, not measured student outcomes.

CBE assumes clearly defined outcomes, aligned assessment and progression based on demonstrated competence [5, 6]. Our findings refine this assumption for EBP. The competency is named in regulations and international frameworks, but participants described no shared breakdown into observable milestones, and limited experience in designing assessments (ST 2.2), particularly for EBP in clinical placements, where competence is ultimately judged. At the same time, the findings challenge a purely standardised reading of CBE: participants valued academic freedom as the space in which they could try new approaches (ST 1.4). This points to a tension between shared outcome standards and educator autonomy that any framework for EBP education in Germany will need to address. Because these interpretations rest on the accounts of 11 educators, they indicate directions for theory rather than tests of it.

Not all of the challenges identified are specific to EBP. Time pressure, financial constraints and staff shortages (ST 2.1), as well as students’ difficulties with academic writing (ST 2.4), are general challenges of academisation that affect the whole curriculum. Other challenges relate specifically to EBP: gaps in educators’ preparation for teaching research methods and EBP (ST 2.2), clinical mentors who do not model EBP (ST 2.3), and the absence of agreed approaches to assessing EBP competencies. The distinction matters, because the general challenges may ease as programmes mature, whereas the specific challenges are likely to require targeted measures.

Academic freedom warrants particular consideration. Participants regarded it as foundational for pedagogical innovation; however, this same freedom may result in variability in the depth and quality of EBP teaching, as several participants noted (ST 1.4). Some mechanisms already limit this variability: the HebStPrV defines competency areas that all programmes must address [20], programme accreditation reviews curricula against these requirements, and the state examination provides a common endpoint. These mechanisms primarily regulate curricular content rather than pedagogical methods, and none currently specify EBP competencies in detail. Developing shared learning outcomes and assessment rubrics for EBP, agreed upon across programmes, could reduce this variation without mandating specific teaching methods. This proposal arises from our interpretation rather than a practice observed in the participating programmes. The interviews confirm that EBP teaching varies between educators and programmes, but do not indicate any particular form of coordination. Whether shared outcomes and rubrics are desirable or feasible remains a topic for discussion among educators, students, clinical partners, and regulatory bodies, followed by evaluation.

### Policy and practice implications

The findings have several implications for midwifery education policy and practice in Germany. These should be regarded as interpretive suggestions derived from this specific qualitative sample, rather than as prescriptive directives.

First, the educator competency gaps identified in Theme 2 (ST 2.2) indicate that structured faculty development focused on EBP teaching may be beneficial. Such development could include short modular courses on teaching research methods, designing active learning and assessment aligned with CBE principles, peer observation of teaching, and communities of practice that connect educators across universities to share materials, building on existing valued networks (ST 3.2). For instance, a semester-long development cycle could involve educators attending a workshop, redesigning one EBP session, being observed by a peer, and collaboratively reviewing student work. These measures would address participants’ reports of abrupt and largely unsupported transitions from clinical to academic roles.

Second, participants’ accounts of the theory-practice divide (ST 2.3) and bridging initiatives at three institutions indicate that formal partnerships between universities and clinical sites could enhance EBP learning beyond the capacity of individual educators. Potential strategies include joint appointments for practice educators, joint journal clubs where students and clinical midwives appraise evidence on practice-related questions, bachelor theses addressing issues raised by clinical partners, and EBP training for clinical supervisors. For example, a joint learning activity could involve students bringing a question from their placement, appraising relevant evidence with both a university educator and a clinical supervisor, and determining its application in the clinical setting. Such activities, similar to the faculty development cycle, should be piloted and evaluated prior to broader implementation. Frameworks for research-inspired teaching [23] and the 4 M model for its implementation [39] provide structured foundations for these initiatives, and international evidence highlights collaborative partnerships as a critical link between teaching, research, and EBP [22].

Third, participants described no nationally coordinated description of EBP competencies, which leaves each programme, and often each educator, to define them independently (ST 1.4). A shared national description of EBP competencies for German midwifery programmes, drawing on the ICM Essential Competencies [4] and the competency areas of the HebStPrV [20], could reduce this variation while preserving educators’ freedom in how they teach. This suggestion arises from a small qualitative sample, however, and its feasibility and value would need to be examined in larger studies involving educators, students, clinical partners and regulatory bodies.

### Limitations

Several limitations should be acknowledged. The most significant for interpretation are the limited transferability beyond the three participating states, the probable selection of educators already engaged with EBP, and the absence of student outcome data, which precludes assessment of the effectiveness of the teaching approaches described. The study was conducted in only three of Germany’s sixteen states, with four of the five institutions located in Bavaria and Lower Saxony. Institutions from North Rhine-Westphalia, northern city-states, and other regions were not included. Variations in governance, resource allocation, and regulations across states may restrict the applicability of these findings. Therefore, transferability should be considered with caution. These findings provide context-specific insights rather than a national perspective. Educators in other states, including eastern and city states, may operate under different conditions, and any national recommendations based on these findings would require validation in a larger and more representative sample.

Second, the sample included only educators in active teaching roles, which may overrepresent positive perspectives compared with colleagues who have left EBP teaching because of barriers. In addition, recruitment relied partly on the professional networks of the PI and on the German Midwifery Academic Society, so educators already engaged with EBP and educational innovation may be overrepresented, while those who find such teaching difficult or are sceptical of it may be underrepresented. Facilitators may therefore be overstated and some barriers understated. We sought to reduce this risk by including educators across roles and with teaching experience ranging from 1 to 19 years, and by asking explicitly about obstacles during interviews; the findings are nevertheless best read as describing educators who are broadly engaged with EBP teaching. The prior professional relationship between the PI and some participants may also have encouraged socially desirable answers, for example by making participants less willing to criticise their own teaching or institution, and may have influenced which accounts the PI found most salient during analysis. To reduce this risk, participants were explicitly invited to give critical responses, the PI kept reflexive memos throughout, a second researcher coded the first 4 transcripts independently, and emerging interpretations were reviewed in peer debriefing with supervisors from different disciplinary backgrounds. The study did not include student or clinical supervisor perspectives, which may differ from educators’ accounts, particularly regarding the theory–practice divide. Recruitment ended because no additional participants could be recruited, rather than on the basis of a prospectively documented saturation threshold. Invitation and non-participation counts and the presence or absence of other people during interviews were not reported, limiting assessment of recruitment and interview conditions.

Third, the study focused on implementation experiences and processes rather than student EBP competency outcomes. The findings illustrate educators’ teaching approaches and the contextual factors influencing them, but do not demonstrate whether these methods improved students’ EBP competency. Qualitative accounts of practice are not a substitute for outcome measurement, as each type of evidence addresses distinct research questions.

Fourth, programme maturity may have shaped the findings. All programmes were established between 2019 and 2021, so data were collected in the early years of programme development, when curricula, staffing and clinical partnerships were still being built. Educators in the programmes established in 2019 described having had more time to build teaching confidence than those in newer programmes (ST 1.1), and staff shortages were most pronounced in newer programmes with smaller faculties (ST 2.1). Some barriers may therefore reflect an early stage of development rather than lasting features of EBP teaching, and the balance of barriers and facilitators may change as programmes mature. Differences in institution type, public or private status, funding and placement arrangements may have contributed further variation. With 2 or 3 participants per institution, the study could not compare institutions systematically, and apparent differences between institutions may reflect individual accounts rather than stable institutional patterns; they should be read as indicative only.

### Future research directions

The findings indicate three sequential priorities for future research. First, replicating the study across a broader geographic and institutional spectrum, including underrepresented federal states and diverse programme-governance models, would determine whether the identified themes are generalizable beyond the current sample. This approach would also facilitate a more systematic comparison of factors that enable or hinder implementation.

Second, addressing the limitation of the current educator-only design, future research should incorporate multistakeholder perspectives, including those of students and clinical supervisors. This broader approach would yield a more comprehensive understanding of EBP competency development across the education–practice continuum.

Third, once a more comprehensive descriptive foundation is established, outcome-oriented research should employ validated EBP competency instruments. These instruments could be adapted for the German academic midwifery context from established tools such as the Fresno Test, EBPQ, or EBP-COQ [40–42]. Such studies would enable evaluation of whether innovative teaching methods produce measurable improvements in competency. Appropriate research designs include longitudinal studies tracking student cohorts over multiple semesters, mixed methods evaluations of specific pedagogical approaches such as problem-based learning or spiral EBP curricula, and comparative analyses of programmes with differing EBP teaching models. Prior to implementation, these instruments would require translation, cultural adaptation, and psychometric validation with German midwifery students. Separate versions for educators or clinical supervisors would also need to be developed.

## Conclusions

The participating German midwifery educators described pedagogical innovation in implementing contemporary EBP teaching following the HebRefG reform. They operate with considerable autonomy, frequently without formal pedagogical training. Nevertheless, they encounter structural barriers, including systemic resource limitations, gaps in educator competencies, and a persistent divide between theory and practice. These challenges may impede the sustained integration of EBP competencies as intended by the reform. Participants identified three areas requiring further development and evaluation: (1) educator development, (2) formal partnerships between universities and clinical sites, and (3) a unified national framework for EBP competencies. While the legislative transition has facilitated pedagogical transformation, the feasibility and value of these proposals require further evaluation. These conclusions are based on contextually rich insights from a qualitative study and should be interpreted within this context.

## Supplementary Information

Supplementary Material 1.

## Data Availability

Anonymised data can be requested from the corresponding author for reasonable inquiries by submitting a written request specifying your research purpose and data requirements. The corresponding author will review requests in accordance with ethics guidelines and respond with further instructions if access is granted.
