# Supplementary material for: Implementing modern teaching approaches for evidence-based practice in German midwifery university education: a qualitative study of educator experiences, facilitators, and barriers
Source: BMC Med Educ. 2026 Nov 6;26:1804. doi: 10.1186/s12909-026-10807-5 (PMC13741687; doi:10.1186/s12909-026-10807-5)
Supplement: Supplementary file 1 [file 12909_2026_10807_MOESM1_ESM.docx]

**Supplementary Material**

The full codebook, including themes, sub-themes, codes and representative extracts, is available on reasonable request. Table 3 summarises the coding structure.

Supplementary Table S2 provides the COREQ 32-item checklist with manuscript location references and reporting limitations.

**Supplementary Table S2. COREQ 32-item checklist.**

Note: COREQ = Consolidated Criteria for Reporting Qualitative Research [Tong A, Sainsbury P, Craig J. Consolidated criteria for reporting qualitative research (COREQ): a 32-item checklist for interviews and focus groups. Int J Qual Health Care. 2007;19(6):349–57]. IDI = in-depth interview; EBP = evidence-based practice. The checklist identifies manuscript locations and distinguishes reported information from items that are not reported.

| **No.** | **COREQ Item** | **Location in Manuscript** |
| --- | --- | --- |
|  | **Domain 1: Research Team and Reflexivity** |  |
|  | **Personal characteristics** |  |
| 1 | Interviewer/facilitator: which author/s conducted the interview? | Methods > Reflexivity; Methods > Data Collection |
| 2 | Credentials: e.g. PhD, MD | Methods > Reflexivity |
| 3 | Occupation at time of study | Methods > Reflexivity (current teaching position; occupation at the time of data collection is not separately reported) |
| 4 | Gender | Not explicitly reported |
| 5 | Experience and training | Methods > Reflexivity |
|  | **Relationship with participants** |  |
| 6 | Relationship established prior to study commencement | Methods > Reflexivity; Methods > Setting and Participants |
| 7 | Participant knowledge of the interviewer (e.g. personal goals, reasons for research) | Methods > Setting and Participants; Methods > Reflexivity (study information and invitation to give candid responses) |
| 8 | Interviewer characteristics (e.g. assumptions, interests in research area) | Methods > Reflexivity |
|  | **Domain 2: Study Design** |  |
|  | **Theoretical framework** |  |
| 9 | Methodological orientation and theory underpinning the study | Methods > Study Design; Methods > Data Analysis |
|  | **Participant selection** |  |
| 10 | Sampling method | Methods > Setting and Participants |
| 11 | Method of approach (e.g. phone, mail, e-mail) | Methods > Setting and Participants |
| 12 | Sample size | Methods > Setting and Participants; Results > Participant Characteristics |
| 13 | Non-participation (refusals/withdrawals) | Methods > Setting and Participants (non-participation counts not reported) |
|  | **Setting** |  |
| 14 | Setting of data collection (e.g. home, workplace) | Methods > Data Collection |
| 15 | Presence of non-participants | Methods > Data Collection (presence or absence of non-participants not reported) |
| 16 | Description of sample (key characteristics) | Results > Participant Characteristics; Table 2 |
|  | **Data collection** |  |
| 17 | Interview guide (questions, prompts; whether piloted) | Methods > Data Collection |
| 18 | Repeat interviews (whether conducted) | Not explicitly reported; Methods > Data Collection describes individual interviews |
| 19 | Audio/visual recording | Methods > Data Collection |
| 20 | Field notes (made during/after interview) | Methods > Data Collection |
| 21 | Duration of interviews | Methods > Data Collection (45–90 minutes) |
| 22 | Data saturation | Methods > Setting and Participants (pragmatic recruitment endpoint; sample adequacy considered using information power) |
| 23 | Transcripts returned to participants for comment/correction | Methods > Methodological Rigour (summary interpretations reviewed; return of full transcripts not reported) |
|  | **Domain 3: Analysis and Findings** |  |
|  | **Data analysis** |  |
| 24 | Number of data coders | Methods > Data Analysis (PI + second researcher) |
| 25 | Description of the coding tree | Table 3 (summary); full codebook available on reasonable request |
| 26 | Derivation of themes (in advance or from data) | Methods > Data Analysis (inductively derived) |
| 27 | Software used to manage data | Methods > Data Analysis (OpenCode v4.03) |
| 28 | Participant checking (feedback on findings) | Methods > Methodological Rigour (review of summary interpretations) |
|  | **Reporting** |  |
| 29 | Quotations presented to illustrate themes | Results (throughout) |
| 30 | Data and findings consistent | Results (throughout) |
| 31 | Clarity of major and minor themes | Results; Table 3 |
| 32 | Discussion of diverse cases or minor themes | Results; Discussion |
